# The importance of the viable but non-culturable state in human bacterial pathogens

**DOI:** 10.3389/fmicb.2014.00258

**Published:** 2014-06-02

**Authors:** Laam Li, Nilmini Mendis, Hana Trigui, James D. Oliver, Sebastien P. Faucher

**Affiliations:** ^1^Department of Natural Resource Sciences, Faculty of Agricultural and Environmental Sciences, McGill UniversitySte-Anne-de-Bellevue, QC, Canada; ^2^Department of Biology, University of North Carolina at CharlotteCharlotte, NC, USA

**Keywords:** VBNC, stress, resuscitation, virulence, human pathogens, biofilm, antibiotic

## Abstract

Many bacterial species have been found to exist in a viable but non-culturable (VBNC) state since its discovery in 1982. VBNC cells are characterized by a loss of culturability on routine agar, which impairs their detection by conventional plate count techniques. This leads to an underestimation of total viable cells in environmental or clinical samples, and thus poses a risk to public health. In this review, we present recent findings on the VBNC state of human bacterial pathogens. The characteristics of VBNC cells, including the similarities and differences to viable, culturable cells and dead cells, and different detection methods are discussed. Exposure to various stresses can induce the VBNC state, and VBNC cells may be resuscitated back to culturable cells under suitable stimuli. The conditions that trigger the induction of the VBNC state and resuscitation from it are summarized and the mechanisms underlying these two processes are discussed. Last but not least, the significance of VBNC cells and their potential influence on human health are also reviewed.

## Introduction

### Characteristics of VBNC cells

Cultivation is one of the most fundamental steps in microbiology, and the plate count technique is one of the standard cultivation methods for the enumeration of viable bacteria (Buck, 1979; Talaro et al., 2002). However, it was first discovered in 1982 that *Escherichia coli* and *Vibrio cholerae* cells could enter a distinct state called the viable but non-culturable (VBNC) state (Xu et al., 1982). Unlike normal cells that are culturable on suitable media and develop into colonies, VBNC cells are living cells that have lost the ability to grow on routine media, on which they normally grow (Oliver, 2000).

Despite their non-culturability on normally permissive media, VBNC cells are not regarded as dead cells because of various dissimilarities. Dead cells have a damaged membrane that is unable to retain chromosomic and plasmidic DNA, while VBNC cells have an intact membrane containing undamaged genetic information (Heidelberg et al., 1997; Cook and Bolster, 2007). The plasmids, if any, are also retained in VBNC cells (Oliver, 2010). While dead cells are metabolically inactive, VBNC cells are metabolically active and carry out respiration (Lleò et al., 2000; Besnard et al., 2002). High ATP level was found in *Listeria monocytogenes* even one year after entering the VBNC state (Lindbäck et al., 2010). Moreover, dead cells do not express genes, while VBNC cells continue transcription and therefore, mRNA production (Lleò et al., 2000). In contrast to dead cells that no longer utilize nutrients, VBNC cells were shown to have continued uptake and incorporation of amino acids into proteins (Lleò et al., 1998).

Although VBNC cells have many general characteristics as a kind of viable cells, they have a lot of physiological and molecular differences from the viable, culturable cells. These differences include cellular morphology, cell wall and membrane composition, metabolism, gene expression, physical and chemical resistances, adhesion properties and virulence potential. In terms of cellular morphology, a reduction in cell size and thus, an increase in the surface area to volume ratio is commonly found in VBNC cells (Rahman et al., 1994; Du et al., 2007), probably as a strategy to minimize energy requirements (Postgate, 1976; Biosca et al., 1996). Apart from cell dwarfing, cell rounding is also found in VBNC cells of many species (e.g., Adams et al., 2003; Cook and Bolster, 2007). *Campylobacter* spp. changes from the characteristic spiral shape in the exponential phase to a coccoid shape in the VBNC state (Thomas et al., 2002). *Burkholderia pseudomallei* and *V. cholerae* cells also changes from rods during exponential growth to cocci in the VBNC state (Inglis and Sagripanti, 2006; Senoh et al., 2010). These morphological changes are commonly found in VBNC cells, however, similar changes are also found in non-VBNC cells that live under stressful conditions, so a change in morphology alone cannot be used as the sole parameter to judge whether the cells are in VBNC state or not (Pinto et al., 2013).

VBNC cells also show marked differences in cell wall and membrane composition, including proteins, fatty acids and peptidoglycan. Rearrangement of the outer membrane subproteome observed in *E. coli* was highly dependent on the conditions used to induce the VBNC state. For example, differential expression of three outer membrane proteins (Omp), antigen 43 β-subunit, TolC, and OmpT, was found in cells exposed to nutrient-limited phosphate buffered saline (PBS), and the most drastic changes showing 106 proteins being modulated were found in those exposed to natural seawater and light (Muela et al., 2008). Significant increases in the percentage of unsaturated fatty acids and fatty acids with less than 16 carbons were found in *V. vulnificus* entering the VBNC state, and significant changes in the percentage of hexadecanoic, hexadecenoic, and octadecanoic acids were found in the VBNC cells (Day and Oliver, 2004). Moreover, an increase in peptidoglycan cross-linking was observed in VBNC cells of *Enterococcus faecalis* (Signoretto et al., 2000).

VBNC cells have a lower metabolic rate (Shleeva et al., 2004) and a different gene expression profile compared to culturable cells growing in exponential phase. The expression of *ompW* was significantly induced in VBNC cells of *E. coli* (Asakura et al., 2008). In VBNC cells of *V. cholerae*, a study showed that 58 genes related to regulatory functions, cellular processes, energy metabolism as well as transport and binding were induced by more than 5-fold (Asakura et al., 2007a), while another study reported a reduction in 16S rRNA and the mRNA level of *tuf*, *rpoS*, and *relA*, genes responsible for protein synthesis and stress responses (González-Escalona et al., 2006).

In general, VBNC cells have higher physical and chemical resistance than culturable cells, which might be due to their reduced metabolic rate and a cell wall strengthened by increased peptidoglycan cross-linking (Signoretto et al., 2000). In terms of physical stress, VBNC cells of *V. vulnificus* are more resistant to mechanical destruction by sonication (Weichart and Kjelleberg, 1996), and those of *Mycobacterium smegmatis* are more resistant to high temperature (Anuchin et al., 2009). In terms of chemical stress, greater tolerance against low salinity and pH, ethanol, chlorine, and antibiotics was observed in VBNC cells of *V. parahaemolyticus* (Wong and Wang, 2004), *V. vulnificus* (Weichart and Kjelleberg, 1996), *Campylobacter jejuni* (Rowe et al., 1998) and *E. faecalis* (Lleò et al., 2007a), respectively. A recent study has shown that *V. vulnificus* in VBNC state has a higher resistance to a variety of challenges, including heat, low pH, ethanol, antibiotic, heavy metal, oxidative and osmotic stress, than those growing in exponential phase (Nowakowska and Oliver, 2013).

At this point it is necessary to differentiate between VBNC cells and persister cells. Persistence refers to the situation where a subpopulation is resistant to an antibiotic, whereas the bulk of the population is sensitive to this antibiotic. The persister cells are viewed as phenotypic variants in the population. To date, persister cells have only been characterized as being in a non-growing state and are antibiotic tolerant (Helaine and Kugelberg, 2014). In fact, VBNC cells have also been shown to be similarly antibiotic tolerant, as well as exhibiting tolerance to heavy metals, high and low temperature, high and low pH, oxidative and osmotic challenges, and ethanol (Nowakowska and Oliver, 2013). Thus, the distinction between “persisters” and “viable but non culturable” cells appears artificial, and while there has been much published in recent years on the former, the literature on the latter is voluminous, and to date there are no convincing studies indicating they are not the same, or at least variants, of the same phenomenon (Wood et al., 2013). The phenotypes and metabolic programs of persister cells have been discussed in a recent review, and will not be covered here (Amato et al., 2014).

VBNC cells can also show changes in adhesion and virulence properties. For example, VBNC cells of *C. jejuni* retain their ability of attachment to stainless steel (Duffy and Dykes, 2009), while *V. cholerae* has a lower rate of adhesion to human intestinal cells (Pruzzo et al., 2003) and *E. faecalis* is unable to attach to plastic surfaces and initiate biofilm formation (Lleò et al., 2007b). *L. monocytogenes* in a VBNC state cannot cause infection (Cappelier et al., 2005; Lindbäck et al., 2010). In *V. vulnificus*, the progressive reduction of virulence is time dependent (Oliver and Bockian, 1995). Nonetheless, some VBNC cells are still virulent and even cause fatal infections, which may be due to rapid resuscitation into culturable cells in suitable hosts (Oliver and Bockian, 1995; Baffone et al., 2003; Du et al., 2007). The alterations in virulence, adhesion as well as resistance of VBNC cells account for their significance to public health (discussed in section Significances).

### The occurrence of VBNC cells and their importance

Many species of bacteria enter the VBNC state when they are exposed to stressful conditions such as starvation and low temperatures (e.g., Biosca et al., 1996; Du et al., 2007), suggesting that this is an adaptive strategy for long-term survival of bacteria under unfavorable environmental conditions (Ducret et al., 2014). This hypothesis is supported by some characteristics of VBNC cells, including higher resistance to exogenous stresses, the ability of long-term survival under stress and the ability of resuscitation. For example, VBNC cells of *V. parahaemolyticus* are more resistant to acidity, allowing them to survive in an adverse environment with low pH (Wong and Wang, 2004). VBNC cells of *V. fluvialis* are viable after six years of starvation, suggesting that bacteria in VBNC state can stay alive over long periods of time, even under continued stress (Amel et al., 2008). More importantly, many species have the ability to resuscitate from the VBNC state back to the culturable state when the stress is removed (e.g., Roszak et al., 1984; Bates and Oliver, 2004). The evidence presented above supports the hypothesis that the VBNC state is a quiescent form of life allowing the organism to wait for suitable conditions to revive (Lleò et al., 2007a). Another hypothesis suggests that the VBNC state is a transitory stage in the degeneration of bacterial population leading to cell death (Thomas et al., 2002; Nyström, 2003). Nonetheless, there is not much evidence supporting the later hypothesis, so the former is generally accepted.

The ability to enter the VBNC state may be advantageous for bacteria, but poses a risk to human health. If VBNC cells are present, the total number of viable bacteria in a sample will be underestimated by the CFU count method due to the inherent non-culturability of VBNC cells. Even worse, if all bacteria in the sample are in VBNC state, the sample may be regarded as germ-free due to non-detection. For bacterial species causing human infections, the underestimation or non-detection of viable cells in quality control samples from the food industry and water distribution systems, or clinical samples may pose serious risks to the public. The risks emerge from the fact that pathogenic bacteria can be avirulent in the VBNC state but regain virulence after resuscitation into culturable cells under suitable conditions (Du et al., 2007). This property of VBNC cells may lead to latency and subsequently, to the recurrence of disease in patients who were thought to be cured (Pai et al., 2000; Rivers and Steck, 2001). Therefore, it is of the utmost importance to understand what species of human pathogens can enter the VBNC state and apply reliable detection methods to quantify the accurate population of viable cells, including both culturable and VBNC cells. Apart from this, the identification of conditions that can induce bacteria to enter VBNC state and the underlying mechanisms, as well as the understanding of resuscitation conditions and mechanisms are necessary to effectively prevent bacterial infections and cure infected patients. In this review, the detection, induction, resuscitation and significance of VBNC cells will be discussed.

## Detection of VBNC cells

The two key properties of VBNC cells are viability and non-culturability, so the presence and abundance of VBNC cells can be determined by comparing the number of viable cells to that of culturable cells in the sample. As a general rule, if the number of culturable cells drops to an undetectable level while the number of viable cells remains high, then the population in the sample has become VBNC cells. Therefore, the first major step for the detection of VBNC cells is the estimation of the remaining culturable cells in the sample by a conventional plate count technique. For this step, it is important to use a rich medium without any additional stress, because some cells that may have been injured during exposure to different VBNC-inducing stresses may be unable to grow on selective or differential media with antibiotics or other stresses. These injured cells have a higher sensitivity to growth medium components that are not normally inhibitory, but they are not regarded as VBNC cells as they are culturable on non-selective media (Pinto et al., 2013). Thus, ensuring the most favorable growth condition allows injured cells to be eliminated from the VBNC population. For instance, after exposure to cold temperature, a population of *V. vulnificus* that normally grows on heart infusion (HI) agar supplemented with 20 μl of 3% hydrogen peroxide (H_2_O_2_) was only able to grow on HI agar with one-tenth concentration of H_2_O_2_ or less (Bogosian et al., 2000). Similarly, under temperature stress and starvation, *C. jejuni* became non-culturable on Karmali agar, which is a selective medium that suppresses the growth of unwanted bacterial species, but it remained culturable on non-selective Columbia blood agar (Cools et al., 2003).

The second major step for the detection of VBNC cells is the estimation of viable cells. One common method involves the use of a differential staining procedure and direct microscopic enumeration. The LIVE/DEAD^®^ *Bac*Light™ assay consists of two fluorescent dyes with different cell permeability characteristics that can be used to differentiate cells with different membrane integrities (Cunningham et al., 2009). The green fluorescent dye, SYTO^®^9, penetrates both intact and damaged membrane and thus, labels all cells. The red fluorescent stain, propidium iodide, can only penetrate damaged membranes and label the injured cells and dead cells (Hurst, 1977). As a result, injured cells and dead cells would appear red under an epifluorescence microscope with suitable filter, while the culturable cells and VBNC cells with an intact membrane would appear green. The concentration of each dye and the appropriate parameters must be empirically validated for each bacterial species using appropriate controls to effectively differentiate between live and dead cells. Flow cytometry can be used with LIVE/DEAD^®^ stains to easily obtain quantitative results (Allegra et al., 2008).

A second method is the detection of gene expression by reverse transcription polymerase chain reaction (RT-PCR). Due to the short half-life of mRNA, a positive signal indicates the presence of mRNA and thus, presence of viable cells that carry out transcription (Adams et al., 2003). As reviewed by Trevors (2011), this method is commonly used in many bacterial species to determine the viability of cells. For example, mRNAs from four housekeeping genes and four virulence genes were detected in a sample of *V. parahaemolyticus* after a 15-day incubation in freshwater at 4°C when the culturability was completely lost, suggesting the presence of VBNC cells (Coutard et al., 2007). The gene expression profile also helps understanding the potential phenotypes of VBNC cells. For example, the *cadF* gene, which encodes an outer membrane protein responsible for fibronectin-binding, was found to be continuously expressed in VBNC cells of *C. jejuni* that maintained the ability of adhesion (Patrone et al., 2013). Some studies have recommended specific genes for the detection of viable cells, such as *rfbE* for *E. coli* (Yaron and Matthews, 2002) and *pbp5* for *E. faecalis* (Lleò et al., 2000).

Other approaches can also be used to detect viable cells. The DNase I protection assay, another molecular method, can distinguish viable cells from dead cells, as only the viable cells have intact membranes to protect genomic DNA from digestion by exogenous nucleases. Using this method, Pawlowski et al. (2011) successfully demonstrated the presence of VBNC cells in a sample of *Yersinia pestis* with an undetectable level of culturable cells.

The direct count of viable bacterial cells (DVC) was first described by Kogure et al. (1979). It was found that viable cells cultured in a rich medium with antibiotics do not replicate but elongate, while dead cells remain unchanged. This method is highly dependent on the antibiotic sensitivity of the bacteria, so the use of nalidixic acid as the antibiotic, as was originally reported, may not be suitable for Gram-positive bacteria and some Gram-negative species (Byrd et al., 1991). Instead, aztreonam can be used for *Cytophaga allerginae* and *Serratia marcescens* (Heidelberg et al., 1997), and ciprofloxacin can be used for *L. monocytogenes* (Besnard et al., 2000). After incubation with antibiotics, acridine orange or 4′,6-diamidino-2-phenylindole (DAPI) can be used to stain the cells and illustrate changes in cellular morphology under microscope (Besnard et al., 2000; Du et al., 2007). Since the mechanisms underlying cell elongation in the presence of antibiotics are not fully understood, and the response of different bacteria to antibiotics may vary (Rice et al., 2000), this method is not commonly used.

Since viable cells, but not dead cells, carry out metabolic reactions and respiration, they can also be detected by the *p*-iodonitrotetrazolium violet (INT) assay based on the activity of electron transport system (Rahman et al., 1994). INT is a soluble tetrazolium salt that can compete with oxygen as the final electron acceptor and be reduced to insoluble formazan in metabolically active cells. Therefore, the formation and accumulation of formazan in cells, which appear as dark red precipitates under microscope, indicate the presence of an active electron transport chain, a characteristic of viable cells (Altman, 1970; Zimmermann et al., 1978). Similarly, another tetrazolium salt, 5-cyano-2,3-ditolyltetrazolium chloride (CTC) or the *Bac*Light™ RedoxSensor™ Green can be used (Besnard et al., 2002; Lahtinen et al., 2008). Moreover, the luciferase assay can be used to estimate ATP generation in viable cells (Lindbäck et al., 2010).

Because viable cells incorporate nutrients, they can also be detected by monitoring the uptake and incorporation of radiolabeled amino acids into protein. ^35^S-labelled methionine,^3^ H-labelled leucine and ^35^S-labelled cysteine/methionine have been used to detect viable cells in *Shigella dysenteriae*, *E. faecalis* and *Y. pestis*, respectively (Rahman et al., 1994; Lleò et al., 1998; Pawlowski et al., 2011).

## Bacteria with a VBNC state

In the past 10 years, several reviews have presented findings of bacteria that can exist in a VBNC state. For instance, Rowan (2004) has focused on foodborne and waterborne bacteria, Oliver (2010) has focused on bacteria that are pathogenic to plants, animals or humans and Pinto et al. (2013) have discussed both pathogenic and non-pathogenic bacteria. Currently, up to 85 species of bacteria have been found to exist in VBNC state in different environmental habitats or experimental conditions. Of these 85 species, 18 species are non-pathogenic and 67 species are pathogenic. The non-pathogenic species were generally found in fermented beverages. Sixteen of the pathogenic species cannot infect humans but can infect other organisms, including plants (Alexander et al., 1999; Grey and Steck, 2001; del Campo et al., 2009), fish (Magariños, Romalde, Barja and Toranzo, 1994; Oliver, 2010), and marine invertebrates such as shrimp (Sun et al., 2008), oysters (Williams et al., 2009), corals (Banin et al., 2000; Israely et al., 2001) and sea urchins (Masuda et al., 2004).

In this review, we specifically focus on bacteria that can cause human infections. Table 1 provides an overview of 51 human pathogens that have been reported to exist in a VBNC state, their inducing conditions, resuscitating conditions and the resuscitation window, if known. The list includes many true pathogens like *E. coli* and *Y. pestis* that cause disease in healthy individuals and may result in fatalities (Gourmelon et al., 1994; Pawlowski et al., 2011). It also includes some opportunistic human pathogens like *Aeromonas hydrophila* and *Agrobacterium tumefaciens* that mainly infect other organisms but also infect immunocompromised patients (Alexander et al., 1999; Rahman et al., 2001). Pathogenic bacteria that can enter a VBNC state have a broad phylogenetic distribution, suggesting that entering VBNC state may be a general strategy adopted by different lineages of bacteria to survive unfavorable conditions.

**Table 1:** **The species of human pathogens with a proven VBNC state**.

**Species**	**VBNC state inducing factor**	**Resuscitation condition**	**Resuscitation window**	**References**
*Acinetobacter calcoaceticus*	Starvation			Lemke and Leff, 2006
*Aeromonas hydrophila*	Starvation	Temperature upshift		Rahman et al., 2001; Maalej et al., 2004
*Agrobacterium tumefaciens*	Starvation, chemicals (copper)			Byrd et al., 1991; Alexander et al., 1999
*Arcobacter butzleri*	Starvation	Rich medium, NOT temperature upshift	270 days	Fera et al., 2008
*Bacillus cereus*	Pulsed electric field			Rowan, 2004
*Burkholderia cepacia*	Starvation			Lemke and Leff, 2006
*Burkholderia pseudomallei*	Low pH, high temperature, osmotic pressure			Reviewed by Inglis and Sagripanti, 2006
*Campylobacter coli*	Starvation, low pH, low temperature	Embryonated chicken eggs, NOT rich medium		Thomas et al., 2002; Chaveerach et al., 2003
*Campylobacter jejuni*	Starvation, low pH, low temperature	Rich medium, rich medium with gas mixture, mouse intestine, embryonated chicken eggs	15 days	Bovill and Mackey, 1997; Thomas et al., 2002; Chaveerach et al., 2003; Cools et al., 2003; Baffone et al., 2006; Cook and Bolster, 2007
*Campylobacter lari*	Starvation, low temperature			Thomas et al., 2002
*Citrobacter freundii*	Starvation, high temperature	Rich medium with/ without enterobacterial autoinducer	11 years	Reissbrodt et al., 2002; Dhiaf et al., 2008
*Cytophaga allerginae*	Aerosolization			Heidelberg et al., 1997
*Edwardsiella tarda*	Starvation, low temperature	Rich medium with temperature upshift, embryonated chicken eggs		Du et al., 2007
*Enterobacter aerogenes*	Starvation			Byrd et al., 1991
*Enterobacter agglomerans*	High temperature	Rich medium with enterobacterial autoinducer		Reissbrodt et al., 2002
*Enterobacter cloacae*				Oliver, 2010
*Enterococcus faecalis (Streptococcus faecalis)*	Starvation, low temperature	Rich medium with temperature upshift, embryonated chick eggs, NOT agar with sodium pyruvate/ beef liver catalase/ superoxide dismutase	60 days	Byrd et al., 1991; Lleò et al., 1998, 2001
*Enterococcus faecium*	Starvation, low temperature	Rich medium, NOT agar with sodium pyruvate/beef liver catalase/ superoxide dismutase	7 days	Lleò et al., 2001
*Enterococcus hirae*	Starvation, low temperature	Rich medium, NOT agar with sodium pyruvate/ beef liver catalase/ superoxide dismutase	60 days	Lleò et al., 2001
*Escherichia coli*	Starvation, light, oxidative stress, high temperature, chemicals (chlorination)	Rich medium with enterobacterial autoinducer, minimal medium with amino acids, supernatant of active growing culture, temperature upshift		Gourmelon et al., 1994; Reissbrodt et al., 2002; Oliver et al., 2005; Cook and Bolster, 2007; Asakura et al., 2008
*Francisella tularensis*				Oliver, 2010
*Haemophilus influenzae*				Ehrlich et al., 2002
*Helicobacter pylori*	Starvation	NOT heat shock, NOT agar with catalase		Adams et al., 2003
*Klebsiella aerogenes*				Oliver, 2010
*Klebsiella planticola*	Aerosolization			Heidelberg et al., 1997
*Klebsiella pneumoniae*	Starvation			Byrd et al., 1991
*Legionella pneumophila*	Starvation, chemicals (disinfectants NaOCl and NH_2_Cl), *Hartmannella vermiformis* supernatant	Amoebae		Steinert et al., 1997; García et al., 2007; Alleron et al., 2008; Buse et al., 2013
*Listeria monocytogenes*	Starvation, low pH, low temperature, low salinity, chemicals (food preservatives), light, pulsed electric field	NOT rich medium with/ without sodium pyvurate		Besnard et al., 2002; Rowan, 2004; Cunningham et al., 2009; Lindbäck et al., 2010
*Micrococcus luteus*	Starvation	Rich medium, supernatant of active growing culture	6 months	Mukamolova et al., 1998b
*Micrococcus varians (Kocuria varians)*				Oliver, 2005
*Mycobacterium smegmatis*	Starvation, oxygen limitation, altered temperature	Rich medium, supernatant of active growing culture, Rpf		Kuznetsov et al., 2004; Shleeva et al., 2004
*Mycobacterium tuberculosis*	Starvation, oxygen limitation	Rich medium with catalase	3.5 months	Downing et al., 2005
*Pseudomonas aeruginosa*	Starvation, low temperature, chemicals (copper)	Temperature upshift, rich medium with copper chelator		Leung et al., 1995; Dwidjosiswojo et al., 2011
*Pseudomonas putida*	Starvation			Lemke and Leff, 2006
*Salmonella enteritidis*	Starvation	Rich medium	<21 days	Roszak et al., 1984
*Salmonella oranienburg*	Starvation, salinity	Rich medium with Rpfs, supernatant from active growing culture		Panutdaporn et al., 2006
*Salmonella typhi*	Starvation			Cho and Kim, 1999
*Salmonella typhimurium*	Starvation, low temperature, light, chemical (chlorination)	Rich medium with enterobacterial autoinducer, heat shock, NOT agar with catalase/ temperature upshift		Caro et al., 1999; Reissbrodt et al., 2002; Gupte et al., 2003; Oliver et al., 2005
*Serratia marcescens*	Aerosolization			Heidelberg et al., 1997
*Shigella dysenteriae*				Oliver, 1995
*Shigella flexneri*				Oliver, 1995
*Shigella sonnei*				Oliver, 1995
*Staphylococcus aureus*				Zandri et al., 2012
*Staphylococcus epidermidis*				Zandri et al., 2012
*Vibrio alginolyticus*				Du et al., 2007
*Vibrio cholera*	Starvation, low temperature	Human intestine, eukaryotic cell lines, rabbit intestine	110 days	Colwell et al., 1985, 1996; Senoh et al., 2010
*Vibrio fluvialis*	Starvation	Rich medium	6 years	Amel et al., 2008
*Vibrio mimicus*				Oliver, 1995
*Vibrio parahaemolyticus*	Starvation, low temperature, low salinity	Temperature upshift	2 weeks	Bates and Oliver, 2004; Wong and Wang, 2004; Wong et al., 2004
*Vibrio vulnificus* (type 1 & 2)	Starvation, low temperature	Rich medium, temperature upshift, mice, clams	3 days	Nilsson et al., 1991; Oliver and Bockian, 1995; Oliver et al., 1995; Biosca et al., 1996; Wong and Liu, 2008
*Yersinia pestis*	Starvation, low temperature	Rich medium		Pawlowski et al., 2011

The inducing factors, resuscitating factors, as well as the longest resuscitation window reported are shown.

There is also broad environmental distribution of human pathogens that exist in a VBNC state. They are found in different kinds of water bodies, including seawater (Maalej et al., 2004; Dhiaf et al., 2008), estuarine water (Oliver et al., 1995), stream water (Lemke and Leff, 2006), lake water (Signoretto et al., 2004), ground water (Cook and Bolster, 2007), tap water (Pawlowski et al., 2011) and drinking water (Byrd et al., 1991). Moreover, VBNC cells of *Salmonella typhimurium* were found in soil (Reissbrodt et al., 2002) and those of *E. coli* were found in processed food (Makino et al., 2000). These findings support the idea that entering the VBNC state may be a common adaptive mechanism of bacteria inhabiting different, stressful environments, instead of a specific mechanism limited to bacteria living in a particular niche (Pinto et al., 2013).

## Induction of the VBNC state

### Conditions

Although VBNC bacteria may remain viable for long periods of time, these cells lose their ability to grow on classical culture media on which they would normally develop into colonies (Oliver, 2005). It was shown that cells enter the VBNC state as a response to an extensive list of both chemically and environmentally unfavorable conditions (Oliver, 2010), including nutrient starvation (Cook and Bolster, 2007), extreme temperatures (Besnard et al., 2002), incubation outside the pH or salinity ranges that are permissive to cell growth (Cunningham et al., 2009), elevated or lowered osmotic concentrations (Asakura et al., 2008; Wong and Liu, 2008), fluctuating oxygen concentrations (Kana et al., 2008; Mascher et al., 2000), exposure to heavy metals (Ghezzi and Steck, 1999; del Campo et al., 2009), exposure to food preservatives (Quirós et al., 2009) and exposure to white light and UV irradiation (Gourmelon et al., 1994). In addition, treatments normally assumed to be bactericidal may instead result in the induction of the VBNC state in a subpopulation, including pasteurization of milk (Gunasekera et al., 2002) and chlorination of wastewater (Oliver, 2005).

To date, no large-scale studies have been performed to compare the effects of diverse conditions on the induction of VBNC state. Such a study is required to provide new information on the conditions that would lead to faster VBNC state inductions. Pinto et al. (2011) suggested that in *E. coli*, the origin of the strains and the incubation temperature are key factors for the speed at which VBNC cells appear in a bacterial population.

### Regulators of the VBNC state

Despite the fact that the formation and the morphological/physiological changes of the VBNC state have been well investigated (Barcina et al., 1997; Colwell and Grimes, 2000), little is known about the genetic control underlying this state. Since, there is a broad range of bacterial species that can enter the VBNC state, it is likely that there is also diversity in the regulatory mechanism. Understanding the molecular control of VBNC state would provide insight into its physiology, and may yield new information to develop novel detection methods or control methods for VBNC bacteria. To our knowledge, no systematic, large-scale screening of mutants for defects in VBNC state induction has ever been performed. Nevertheless, the regulators RpoS and OxyR seem to be important for the induction of VBNC state and are discussed below.

#### RpoS

A simple approach to identify VBNC genes consists of testing the influence of genes known to be involved in the response to other stressful environmental conditions, upon entry into VBNC state and during the survival of VBNC cells (McDougald et al., 2001). This led Boaretti et al. (2003) and Kusumoto et al. (2012) to analyse the involvement of the major stress regulator RpoS, a sigma factor essential for survival in the stationary phase and the general stress response (Lange and Hengge-Aronis, 1991; Hengge-Aronis, 1993). Indeed, RpoS depletion resulted in faster VBNC state induction in *E. coli* and *Salmonella* spp. (Kusumoto et al., 2012). The parental strain of *E. coli* became non-culturable in 33 days in an artificial oligotrophic medium incubated at 4°C, whereas the *rpoS* mutant lost its culturability in only 21 days (Boaretti et al., 2003). Moreover, lack of *rpoS* resulted in a decreased ability to stay in VBNC state for long periods of time, resulting in faster cell death (Boaretti et al., 2003). Smith and Oliver (2006) reported that *V. vulnificus* continues to express *rpoS* in the VBNC state, suggesting that the stress-related genes regulated by *rpoS* are also likely to be expressed in VBNC state and lead to cross-protective effects. For example, once the bacteria encounter a stress, they produce a variety of proteins that serve to enhance survival under other stresses (Rangel, 2011).

In *E. coli*, RpoS mediates the expression of 10% of the genome upon exposure to stressful conditions (Gentry et al., 1993). It has been shown that induction of the stress response governed by RpoS involves the production of the alarmone guanosine tetraphosphate or pentaphosphate, ppGpp and pppGpp respectively [termed together as (p)ppGpp], and results in an increase in the amount of RpoS (Gentry et al., 1993). (p)ppGpp is a general indicator of the nutritional status of the cell and it lies on top of the regulatory network (Cashel et al., 1996). In Beta- and Gammaproteobacteria, intracellular levels of (p)ppGpp are modulated by the RelA and SpoT proteins. RelA is a monofunctional alarmone synthase, and SpoT is a bifunctional synthase/hydrolase (Potrykus and Cashel, 2008). Both RelA and SpoT enzymes can synthesize (p)ppGpp, whereas SpoT can also hydrolyze it (Chatterji et al., 1998). It has been shown that, like the *rpoS* mutant, lack of (p)ppGpp production resulted in faster VBNC state induction (Boaretti et al., 2003). Overproducers of (p)ppGpp displayed viability comparable to that of Δ*rpoS* complemented strains or the wild-type strain (Boaretti et al., 2003). These findings suggest that the level of (p)ppGpp may play a crucial role in the accumulation of RpoS, ultimately leading to enhanced stress resistance in VBNC cells. In *V. cholerae*, the entry into VBNC state changes the expression of *relA* which, in turn, modulates the (p)ppGpp level (González-Escalona et al., 2006; Asakura et al., 2007a). This may lead to further modulation of several major systems, including stress-response, replication and virulence systems. It is noteworthy that ppGpp also play a role in the production of persister cells as well (Amato et al., 2014).

#### OxyR

Cuny et al. (2005) suggested that reactive oxygen species (ROS) play a role in the formation of VBNC cells. When *E. coli* was subjected to H_2_O_2_ treatment, they entered the VBNC state (Arana et al., 1992). In addition, VBNC *E. coli* shows decreased superoxide dismutase activity, resulting in increased oxidative damage (Desnues et al., 2003). This suggests that regulation of the oxidative stress response is involved in the induction of VBNC state.

OxyR is a LysR-type transcriptional regulator and was first identified in *S. typhimurium* (Christman et al., 1985). It has a characteristic N-terminal DNA-binding domain, and is known to regulate oxidative stress-related genes (Tao et al., 1991; Wei et al., 2012). In *V. vulnificus*, mutation of *oxyR* results in loss of culturability after exposure to cold temperature, which can be relieved by cultivation under anaerobic conditions or with an exogenous supply of catalase (Kong et al., 2004). This study attributed the cold-induced loss of catalase activity to the induction of VBNC state, explaining why *Vibrio* spp. are almost undetectable during the winter months but re-emerge during the summer months, when temperature and nutrient level in seawater increase. In addition, two recent studies have investigated the role of two antioxidative enzymes, the alkyl hydroperoxide reductase subunit C (AhpC) (Wang et al., 2013) and the glutathione S-transferase (GST) (Abe et al., 2007), in the induction and maintenance of the VBNC state in *V. parahaemolyticus* and *V. vulnificus*, respectively (discussed in section Effectors Related to the Oxidative Stress Response). Knowing that OxyR regulates the expression of *ahpC* in several Gram-negative bacteria in response to elevated ROS levels (Charoenlap et al., 2005; Hishinuma et al., 2006), and more recently, the expression of *gst* (*Abgst01*) in *Acinetobacter baumannii* in the presence of organic peroxides (Longkumer et al., 2014), we suggest that OxyR may be involved in the regulatory pathway leading to the induction of VBNC state. Direct evidence, such as quantification of ROS and antioxidative activities during the induction of VBNC state in the wild type and the *oxyR* mutant, is needed to further define the role of ROS in this state.

### Effectors of the VBNC state

#### Effectors related to the metabolic pathways to obtain energy

One line of research involves detecting proteins present exclusively in cells in the VBNC state (Heim et al., 2002; de Angelis and Gobbetti, 2004). Heim et al. (2002) studied changes in the proteome of *E. faecalis* in VBNC state after a 20-day incubation in lake water at 4°C. Significant differences in protein profiles were observed among the exponentially growing cells, starved cells and VBNC cells. The proteomic analysis showed a significant down regulation of important proteins during the VBNC state: putative proteins enolase (glycolysis), ATP synthase (oxidative phosphorylation) and a homolog of the *Staphylococcus aureus* elongation factor EF-Tu (involved in protein synthesis, cell growth regulation and stress response). The repression of these genes seems in agreement with the decrease in metabolic activity observed in VBNC cells. On the other hand, three proteins were over-expressed in VBNC cells: a putative catabolite regulator protein (CAA09491), a homolog of the *Listeria innocua* elongation factor EF-Ts, and a homolog of the *Streptococcus pneumoniae* fructose bisphosphate aldolase. In this study, the genetic pathways of *E. faecalis* underlying the VBNC response appear to be, in part, the same as those leading to the starvation response, as indicated by the presence of similar expression profiles for certain proteins like enoyl-ACP reductase (phospholipid biosynthesis). Nonetheless, significant differences were also observed in the protein profiles between starved cells and VBNC cells. A protein homologous to the mannose-specific phosphotransferase system (PTS) of *Clostridium acetobutylicum* was only up-regulated in starved cells, and an unidentified protein (30 kDa at pI 6) was only detected in VBNC cells. While the specific function of this unidentified protein is still unclear, a comparison of 2D gels obtained from different cell states suggests that it may be a modified form of enoyl-ACP reductase.

In *E. coli* O157:H7, entry into the VBNC state was observed after 48 h of incubation in PBS containing 0.05% H_2_O_2_. Under this condition, 11% of the population maintained its membrane integrity. Incubation for 72 h led to a significant reduction in the number of cells with an intact membrane (Asakura et al., 2007b). The elongation factor EF-Tu, which was under-expressed in *E. faecalis* (Heim et al., 2002), maintained its expression level in *E. coli*, suggesting the maintenance of protein synthesis in the latter (Asakura et al., 2007b). The differences in the expression level of elongation factors between *E. coli* and *E. faecalis* suggest that the species as well as stress conditions are key factors in determining the mechanism involved in the induction of VBNC state.

A second line of research involves comparing the global transcription pattern of VBNC cells with that of cells grown in a rich medium (Asakura et al., 2007a,b). The transcriptomic analysis of *V. vulnificus* VBNC cells following incubation in artificial seawater at 4°C for 70 days has highlighted the induction and repression of hundreds of genes compared to stationary phase cells (Asakura et al., 2007a). The genes whose expression is significantly affected in the VBNC state are classified into the following functional groups: protein synthesis, energy metabolism, cell envelope, cellular processes, regulatory function, amino acid synthesis, and transport proteins. The genes expressed in the VBNC state include those involved in metal ion (iron, magnesium, potassium and cobalamin) transportation, chemotaxis and motility, pilus assembly and chitin utilization. All together, these findings indicate that both the proteomic and transcriptomic profiles of VBNC cells are different from those of cells that are either starved or exponentially growing. This demonstrates that the VBNC state constitutes a physiologically distinct state within the life cycle of a bacterium, which is activated in response to environmental stresses.

#### Effectors related to the oxidative stress response

The involvement of antioxidative factors in the VBNC state has been investigated (Bogosian et al., 2000; Wai et al., 2000). The addition of catalase or other ROS scavengers, such as sodium pyruvate, to the culture medium improves the culturability of VBNC cultures of *A. hydrophila* (Wai et al., 2000), *E. coli* (Mizunoe et al., 1999), and *V. vulnificus* (Bogosian et al., 2000).

Recently, Wang et al. (2013) investigated the antioxidative activities of alkyl hydroperoxide reductase subunit C (*ahpC1* and *ahpC2*) against H_2_O_2_ and organic peroxide in *V. parahaemolyticus* in the context of the VBNC state and found that *ahpC2* alone played a significant role in the induction and maintenance of the VBNC state at 4°C. While an earlier study found an enhanced quantity of AhpC2 protein in the VBNC cells (Lai et al., 2009), the latest study showed that the transcripts of both *ahpC1* and *ahpC2* decreased to low levels by approximately three orders of magnitude during the induction of the VBNC state (Wang et al., 2013). The protective function of AhpC2 at 4°C was higher than that of AhpC1. Indeed, the time required to induce and maintain the VBNC state at 4°C in a modified Morita mineral salt solution with 0.5% NaCl in an *ahpC2* mutant and an *ahpC1ahpC2* double mutant was significantly shorter than that for the parental strain and the *ahpC1* mutant (Wang et al., 2013). Complementation with an *ahpC2* gene reversed the effects of the *ahpC2* mutation by increasing the time required for induction and maintenance of the VBNC state (Wang et al., 2013).

GST is another protein related to the oxidative stress response and is also involved in the VBNC state. It is a cytosolic protein that detoxifies endogenous compounds, such as peroxidized lipids, by conjugation with reduced glutathione (Marrs, 1996). In 2007, Abe et al. showed that a mutation causing over-expression of GST was responsible for the suppression of the ability to enter the VBNC state in *V. vulnificus* (Abe et al., 2007). Since GST is linked to the antioxidative response, the authors hypothesized that the induction of VBNC state is directly connected to oxidative stress, and that removing this stress would abolish the entry into VBNC state. This hypothesis was supported by Oliver (2010), who showed an important role of the *V. vulnificus* catalase KatG in its VBNC state. In this bacterium, expression of *katG* is repressed upon the entry into VBNC state, but is induced when cells exit the VBNC state and become culturable again.

#### Effectors related to the outer membrane proteins

In Gram-negative bacteria, the outer membrane is an important physical and functional barrier separating the inside of cells and their surrounding environment. It consists of phospholipids, lipopolysaccharides and outer membrane proteins (OMPs) (Koebnik et al., 2000). Some OMPs, called porins, allow the passive diffusion of small, charged and uncharged molecules into bacterial cells (Nikaido and Vaara, 1985). In *E. coli*, the major outer membrane proteins are OmpF and OmpC (Misra and Reeves, 1987). Changes in the amount of OMPs in response to environmental stimuli have a major consequence on the survival of *E. coli* in stressful conditions, including starvation, changes in osmolarity and the VBNC state (Nikaido and Vaara, 1985; Özkanca and Flint, 2002; Darcan et al., 2009). In *E.* coli, the highest number of cells entering the VBNC state was found in an *ompCompF* double mutant strain compared to the wild-type and single mutants (Darcan et al., 2009). This is consistent with the phenotype of the *ompCompF* double mutant in *S. typhimurium* that lost culturability and entered the VBNC state when subjected to oxidative stress (Ozkanca et al., 2002).

The EnvZ/OmpR system regulates expression of the OmpF and OmpC proteins (Russo and Silhavy, 1991). OmpR is a cytoplasmic DNA-binding protein (Aiba et al., 1989). EnvZ is an environmental sensor bound to the inner membrane and has both kinase and phosphatase activities (Forst et al., 1987; Igo and Silhavy, 1988). Darcan et al. (2009) showed that the loss of the EnvZ protein has no effect on survival, but prevents the organism from sensing the changes in environment and thus, interfering with the entry into VBNC state. Indeed, *envZ* mutants were found not to enter the VBNC state and stayed culturable for a longer period of time. Nonetheless, it is not clear if this phenotype is specific to the VBNC induction condition which, in this case, was osmotic stress.

## Resuscitation of VBNC cells

### Determination of the resuscitation

It is important to note that bacteria that enter the VBNC state may become culturable again, and thus this state may be reversible. The term “resuscitation” was first used by Roszak et al. (1984) to describe the recovery of non-culturable cells of *S. enteritidis* subsequent to the addition of HI broth. Two decades later, Baffone et al. (2006) defined resuscitation as the reversal of metabolic and physiological changes that characterize VBNC cells. Of the species of human pathogens that can enter the VBNC state, resuscitation has been reported in only 26 species (17 genera) (Table 1). Resuscitation of these species was triggered by a variety of stimuli, such as an increase in temperature, increase in nutrients concentration, and the presence of host cells (discussed in section Factors Stimulating Resuscitation).

The first roadblock that researchers encountered when performing resuscitation studies is the difficulty to differentiate between the resuscitation of VBNC cells and the normal growth of residual culturable cells in a sample. To date, there are no readily available methods to distinguish between culturable cells that arise from resuscitation and those from normal growth after exposure to the stimuli. Therefore, the ideal timing to study resuscitation is when there are no more culturable cells present in the sample. Although the complete absence of culturable cells cannot be guaranteed because of the detection limits, resuscitation experiments are usually conducted after the number of culturable cells drops to an undetectable level, while the number of VBNC cells remains high (Biosca et al., 1996; Cappelier et al., 1999; Chaveerach et al., 2003; Downing et al., 2005). Some studies have carried out extra steps to minimize the number of culturable cells in the sample. For instance, Whitesides and Oliver (1997) performed serial dilutions to further reduce the proportion of culturable cells, and Lleò et al. (1998) used minimal inhibitory concentrations of antibiotics to kill the remaining culturable cells, but not the more resistant VBNC cells before starting resuscitation. Nonetheless, the use of antibiotics is not recommended, as it was found that antibiotics can prohibit the resuscitation of *E. faecalis* from the VBNC state by influencing cell wall biosynthesis (Lleò et al., 2007a).

### Factors affecting resuscitation

Resuscitation has only been proven in half of the human pathogens that exist in the VBNC state, but this does not mean the others cannot be resuscitated at all. It is likely that the resuscitation requirements for the latter were never met. For example, Chaveerach et al. (2003) were able to resuscitate *C. jejuni* by incubating the VBNC cells in embryonated chicken eggs but not in rich medium, showing that the condition or stimulus required for resuscitation could be very specific. In fact, many factors can influence the successfulness of resuscitation, such as the strain used, the age of VBNC cells, the conditions that induced the VBNC state and, of course, the conditions provided for resuscitation (Pinto et al., 2011). A comprehensive study done by Pinto et al. (2011) clearly demonstrated the effects of these four factors on the resuscitation of *E. coli*. Their study showed that different strains of *E. coli* in a VBNC state can be resuscitated in different media, indicating that the resuscitation process is highly dependent on the strains and resuscitating conditions. It was also found that resuscitation only occurs within a limited period of time after entry into the VBNC state, so it is dependent on the age of VBNC cells. Furthermore, this study demonstrated that cells that were induced into a VBNC state at 4°C could be resuscitated by adding rich media, but those induced at room temperature could not be resuscitated by any of the 41 media tested, indicating that resuscitation also depends on the inducing conditions. Apart from the direct effects on resuscitation, the strain of bacteria may also interact with other factors regulating resuscitation. For example, the strain of *V. parahaemolyticus* can affect the period of time that the VBNC cells remain resuscitable (Wong et al., 2004), and the strain of *S. typhimurium* can affect the conditions required for resuscitation (Reissbrodt et al., 2002).

Pinto et al. (2013) first introduced the term “resuscitation window,” which is defined as the period of time in which VBNC cells maintain their ability to resuscitate under suitable stimuli. When exposed to the inducing condition, different cells in a sample enter a VBNC state at different times. It was hypothesized that VBNC cells of the same species have a fixed resuscitation window, so the older VBNC cells will lose their resuscitation ability earlier than the younger VBNC cells, resulting in a reduction of total resuscitable cells over time. This is supported by Senoh et al. (2010), who reported a gradual reduction in the number of resuscitable cells from 74 to 91 days after *V. cholerae* entered a VBNC state. A similar reduction in the number of resuscitable cells over time in a VBNC population was also observed in *E. faecalis* and *E. hirae* (Lleò et al., 2001).

In most published studies, the cells' ability of resuscitation is tested right after the whole population has become VBNC. Therefore, not much information is available on the length of resuscitation window of different bacteria. Some studies report the successful resuscitation of VBNC cells at a particular age but do not repeat the resuscitation experiment at later time points, so the reported resuscitation window may be underestimated (Oliver and Bockian, 1995; Downing et al., 2005; Amel et al., 2008). There are only a few studies that have tried to determine the exact resuscitation window by studying the resuscitation of VBNC cells at different ages. Fera et al. (2008) investigated the VBNC cells of *Arcobacter butzleri* at four different ages and deduced that cells younger than 270 days can be resuscitated by addition of rich medium. Mukamolova et al. (1998b) found that 3-month-old and 6-month-old VBNC cells of *Micrococcus luteus* can be resuscitated within two months and 10 days, respectively, suggesting that the resuscitation window of this species is around six months. According to the available information, there is great variation in the resuscitation windows between different species (Table 1), ranging from 4 days in *S. enteritidis* (Roszak et al., 1984) to 11 years in *Citrobacter freundii* (Dhiaf et al., 2008).

### Factors stimulating resuscitation

Rich medium was the first stimulus found to resuscitate VBNC cells in *S. enteritidis* (Roszak et al., 1984). Since then, additional studies have examined a variety of stimuli that can trigger resuscitation. It was found that resuscitation can be mediated by a physical stimulus like temperature upshift (Maalej et al., 2004), and different kinds of chemical stimuli including gas mixtures (Bovill and Mackey, 1997), amino acids (Pinto et al., 2011), rich media (Amel et al., 2008), supernatant from actively growing cultures (Mukamolova et al., 1998b) or compounds secreted by actively growing cells (Reissbrodt et al., 2002; Panutdaporn et al., 2006), as well as the presence of host cells (Chaveerach et al., 2003). As mentioned previously, these stimuli may not be applicable to resuscitate all species or even the same species in different trials because of other interacting factors. For instance, temperature upshift can resuscitate VBNC cells of *Pseudomonas aeruginosa* (Leung et al., 1995) but not *A. butzleri* and *Helicobacter pylori* (Adams et al., 2003; Fera et al., 2008). Rich medium resuscitated *C. jejuni* in the study of Cools et al. (2003) but not in other studies (Chaveerach et al., 2003; Cook and Bolster, 2007). Among the human pathogens, *C. jejuni*, *E. coli*, and *V. vulnificus* are the three species that have been resuscitated by the greatest variety of stimuli (Table 1).

Interestingly, several studies have reported the unsuccessful resuscitation of VBNC cells after adding sodium pyruvate, catalase or superoxide dismutase onto agar plates (Lleò et al., 2001; Adams et al., 2003; Gupte et al., 2003; Lindbäck et al., 2003). These antioxidants can neutralize or prevent the formation of ROS such as H_2_O_2_ in the medium. These findings further prove that VBNC cells, in contrast to injured cells, do not lose their culturability solely because of sensitivity to ROS (Lleò et al., 2001). Some studies seem to contradict this view, showing that non-culturable cells can be recovered when agar plates are supplemented with ROS scavengers. For example, *S. typhimurium* was recovered by ferrioxamine E and oxyrase (Reissbrodt et al., 2002), *V. vulnificus* was recovered by catalase and pyruvate (Oliver, 2010), and *Legionella pneumophila* was recovered by pyruvate and glutamate (Ducret et al., 2014). However, further analysis of these studies show that the antioxidants only recovered a subpopulation (P1) of the non-culturable cells. In *S. typhimurium*, another subpopulation (P2) of non-culturable cells, which could not be recovered by supplementing with antioxidants, was resuscitated by another well-known resuscitation stimulus, an autoinducer (discussed in section Mechanisms of Resuscitation). These results suggest that P1 is composed of injured cells and P2 is composed of VBNC cells, so the increase in the number of culturable cells on antioxidant-supplemented agar was probably due to the recovery of injured cells instead of the resuscitation of VBNC cells.

### Mechanisms of resuscitation

#### Host cells

The mechanisms underlying the resuscitation of VBNC cells remain largely unknown, especially for those triggered by host cells due to complicated bacteria-host interactions that are likely to play a major role. Bacteria in the VBNC state have been found to be resuscitated by amoebae (García et al., 2007), eukaryotic cell lines (Senoh et al., 2010), clams (Birbari et al., 2000), embryonated chicken eggs (Cappelier et al., 1999), mice (Baffone et al., 2006), rabbits (Colwell et al., 1985) and human volunteers (Colwell et al., 1996). These cells or animals are typically the natural host of the resuscitated bacteria. For example, the two species of amoeba, *Acanthamoeba castellanii* and *A. polyphaga*, that were shown to resuscitate VBNC cells of *L. pneumophila*, are the natural hosts of this bacterium (Steinert et al., 1997; García et al., 2007). Within the list of potential hosts, embryonated chicken egg can resuscitate the most species of VBNC cells, including *Campylobacter coli* (Chaveerach et al., 2003), *C. jejuni* (Cappelier et al., 1999), *Edwardsiella tarda* (Du et al., 2007) and *E. faecalis* (Lleò et al., 1998). This may be because of the high nutrient content of the yolk sac and/or the warm temperature during incubation.

#### Removal of stress and presence of specific compounds

Apart from resuscitation triggered by host cells, two requirements were proposed as being crucial for the occurrence of resuscitation. The first is the removal of external stress. It is known that bacteria enter a VBNC state under stressful conditions such as starvation and cold temperature, the two most common inducers (Table 1). Therefore, the addition of rich medium and/or the upshift of incubating temperature may allow the resuscitation of VBNC cells through elimination of the existing stress. Optimal medium concentration and incubation temperature are required for successful resuscitation. For starvation-induced VBNC cells of *M. luteus*, the proportion of cells being resuscitated depends strongly on the concentration of yeast extract in the medium. The optimal concentration depends on the age of VBNC cells, and a high concentration of yeast extract may damage the membranes of resuscitated cells, and consequently, affect their culturability (Mukamolova et al., 1998b). Similar to medium concentration, a temperature that is too high may prohibit resuscitation, as VBNC cells of *V. parahaemolyticus* that were induced by low temperature could be resuscitated at 22°C but not at 37°C (Wong et al., 2004). In contrary, more resuscitation of VBNC *E. coli* was observed at 37°C than at 25°C (Pinto et al., 2011), suggesting that the optimal temperature is species-dependent. Another evidence supporting the importance of stress removal in resuscitation was presented by Dwidjosiswojo et al. (2011). In their study, the VBNC cells of *P. aeruginosa* that were induced by copper ions were fully resuscitated in a solution containing the copper chelator diethyldithiocarbamate within 14 days.

The second hypothesis explaining resuscitation triggers is the presence of specific compounds as a signal. The compounds that were found to resuscitate VBNC cells include amino acids, resuscitation-promoting factors (Rpfs) and autoinducers (Mukamolova et al., 1998a; Reissbrodt et al., 2002; Pinto et al., 2011). Pinto et al. (2011) proposed that the resuscitation of VBNC cells is somehow similar to the germination of dormant spores, which can be triggered by specific amino acids (Atluri et al., 2006). Therefore, they tested the ability of VBNC *E. coli* to resuscitate under a minimal medium supplemented with different amino acids, and found that a combination of leucine, glutamine, methionine and threonine would be sufficient to trigger resuscitation of strain Eco3 (Pinto et al., 2011). It was suggested that these amino acids may bind to receptors on the cell surface or be transported into the cells to initiate resuscitation (Pinto et al., 2013).

***Rpfs***. Rpf was first described by Mukamolova et al. (1998a) as a protein produced by *Micrococcus luteus* that can stimulate bacterial growth by reducing the lag phase and resuscitating VBNC cells in picomolar concentrations. This extracellular protein has been purified from the supernatant of *M. luteus* cultures growing in lactate minimal medium. It was found to have cross-species activity as it can affect the growth of five different *Micrococcus* species. Shleeva et al. (2004) have demonstrated the importance of Rpf by various methods. They successfully resuscitated the VBNC cells of *Mycobacterium smegmatis* by adding recombinant Rpf protein produced by *M. luteus*, co-culturing with *M. luteus* that secretes Rpf, as well as inserting a plasmid containing the *M. luteus rpf* gene for endogenous synthesis of Rpf. This study further suggested that *M. smegmatis* can produce its own Rpf-like protein, as the supernatant from a growing culture was able to resuscitate its VBNC cells.

In contrast to these two species, *Mycobacterium tuberculosis* produces five Rpf-like proteins, which are encoded by *rpfA* to *rpfE*. These genes were thought to be functionally redundant and dispensable for growth, as the proteins seemed to have similar characteristics and properties (Mukamolova et al., 2002) and the deletion of all five genes did not affect the growth in rich medium (Kana et al., 2008). However, these five *rpf* genes were expressed differently during exponential growth, exposure to stress and early resuscitation, suggesting they play different roles in bacterial growth and survival (Gupta et al., 2010). Moreover, the deletion of three *rpf* genes prohibited the growth in mice as well as the resuscitation, demonstrating their functions cannot be compensated by the other two *rpf* genes, which were still present in this mutant strain (Downing et al., 2005). Kana et al. (2008) also reported that different combinations of *rpf* gene deletions result in distinct changes to colony formation on agar plates, sensitivity to the detergent sodium dodecylsulphate and virulence in mice. All these findings suggest that the five Rpf proteins produced by *M. tuberculosis* are only partially redundant.

Three models have been proposed to explain the mechanism underlying Rpf-mediated resuscitation (Pinto et al., 2013). The first model suggests that Rpfs are cell-signaling molecules that are secreted by actively growing cells which can bind to the cell surface receptors on VBNC cells to initiate resuscitation. This model was first proposed by Mukamolova et al. (1998a) as Rpf, similar to other cell-signaling molecules, can stimulate cell growth and is likely to be involved in the control of cell replication.

The second model suggests that, instead of binding to receptors, Rpfs degrade or remodel the cell wall peptidoglycan of VBNC cells, thereby triggering resuscitation. Although no studies have demonstrated the molecular mechanisms underlying resuscitation triggered by peptidoglycan alteration, this model is supported by the fact that all Rpfs contain a conserved domain that is highly homologous to lysozyme and transglycosylase, which are both known to degrade the peptidoglycan of bacterial cell walls (Cohen-Gonsaud et al., 2005). Moreover, RpfB and RpfE were found to interact with Rpf-interacting protein A (RipA), which is a peptidoglycan hydrolase (Hett et al., 2007; Kana et al., 2008). The ability of Rpfs to degrade peptidoglycan via hydrolysis was recently demonstrated (Mukamolova et al., 2006), and this ability seems to be responsible for the resuscitation of *M. smegmatis* (Telkov et al., 2006). This suggests that the alteration of cell wall peptidoglycan may be crucial for the resuscitation of VBNC cells. It is noteworthy that the modification of the peptidoglycan layer was previously found to be involved in the induction of the VBNC state (Signoretto et al., 2000).

The third model is also based on the cleavage of peptidoglycan by Rpfs. However, instead of direct remodeling of the cell wall of VBNC cells, it suggests that the Rpfs cleave the peptidoglycan layer of the Rpf-producing cells and release small peptidoglycan fragments that bind to cell surface receptors of VBNC cells, thereby triggering resuscitation. This model was proposed because previous studies showed that some Rpfs are bound to the cell wall of Rpf-producing cells instead of being released into the growth medium (Mukamolova et al., 1998a; Koltunov et al., 2010). It was subsequently found that peptidoglycan fragments, either generated from Rpf digestion or ultrasonication, can stimulate the resuscitation of *Micrococcus* spp. (Nikitushkin et al., 2013). PknB is a Ser/Thr membrane kinase with an extracytoplasmic domain that binds peptidoglycan fragments. This protein may be responsible for the resuscitation mechanism, as it was demonstrated that a synthetic muropeptide with high affinity to PknB had a moderate effect on resuscitation (Mir et al., 2011). This model may also provide clues about the mechanism underlying resuscitation in animal models, since lysozymes produced by the immune system damage the peptidoglycan layer and could lead to the release of peptidoglycan subunits.

***Autoinducers***. Unlike the Rpfs that were only described in *Micrococcus* and *Mycobacterium* spp., heat-stable autoinducers are produced by a range of Gram-negative bacteria and some Gram-positive bacteria. They were first found in cultures of *E. coli* growing in serum-SAPI medium supplemented with the human catecholamine hormone, norepinephrine (Freestone et al., 1999). Two years later, they were also found in cultures growing in four other kinds of media without the addition of norepinephrine (Freestone et al., 2001). Apart from thermal stability, these autoinducers are dialyzable, acid- and alkali-stable as well as protease-resistant (Weichart and Kell, 2001). *E. coli* were found to produce at least two kinds of autoinducer, AI-2 and AI-3 (Sperandio et al., 2003), which enter the target cells via a TonB-dependent receptor (Freestone et al., 2001). In *V. vulnificus*, the addition of a LuxR inhibitor has been shown to delay the resuscitation mediated by AI-2, suggesting that this autoinducer is sensed by the master quorum sensing regulator SmcR (a LuxR homolog) (Ayrapetyan et al., 2014). In the same study, the authors found that RpoS is also important for AI-2 mediated resuscitation, and proposed that increased level of AI-2 stimulates *rpoS* expression through the action of LuxR, leading to resuscitation.

Similar to Rpfs, autoinducers can stimulate bacterial growth by reducing the lag phase (Freestone et al., 1999). In addition, they can be extracted from the supernatants of bacterial cultures and initiate resuscitation of VBNC cells of other bacterial species, demonstrating cross-species activity. It was found that the autoinducers from *E. coli* can resuscitate its own VBNC cells (Pinto et al., 2011), while the enterobacterial autoinducers from *Y. ruckeri* can resuscitate four species including *C. freundii*, *E. coli*, *Enterobacter agglomerans*, and *S. typhimurium* (Reissbrodt et al., 2002).

#### Uncertainties waiting to be resolved

The production of Rpfs and autoinducers by bacteria and the importance of these factors in resuscitation have raised another question: Does the resuscitation of VBNC cells require external stimuli or simply rely on factors produced by themselves or other bacterial species? Epstein (2009) proposed that bacteria can awake randomly without relying on environmental stimuli. According to this hypothesis, VBNC cells may resuscitate randomly, dying if the conditions are still unfavorable, or surviving if the conditions are permissive for growth. If the cells survive, they may divide to start a new population and/or secrete a signal to resuscitate other VBNC cells. Interestingly, a recent study have found that the level of autoinducer AI-2 in *V. vulnificus* reaches a peak 5 h after temperature upshift (a resuscitation-stimulating environmental stimulus), but the resuscitation of VBNC cells only became detectable an additional 2 h later (Ayrapetyan et al., 2014). The authors agreed to Epstein's hypothesis and suggested that resuscitation of the major population occurs after sensing the signal produced by a small, undetectable population of cells that resuscitated stochastically. Another possibility is that the signal is produced by cells remaining in VBNC state in response to a permissive environment, but no supporting or refuting evidences of this theory have ever been shown. Even if the signals have been proven to be produced by resuscitated cells, further investigation is still required to validate Epstein's hypothesis, as the current findings cannot confirm whether an undetectable number of cells resuscitated randomly before, or particularly after, the environment become suitable. The only conclusion that can be drawn is that particular environmental condition (e.g., temperature upshift or nutrient availability) is the prerequisite for the production of Rpfs or autoinducers and the resuscitation of the major population of VBNC cells.

Since the discovery of VBNC cells in the 1980s, few studies have investigated the molecular mechanisms underlying the process of resuscitation. It is known that the expression of *ompW* can regulate resuscitation in *E. coli* (Asakura et al., 2008) and the expression of *luxS* can affect AI-2 production and by consequence, resuscitation in *V. vulnificus* (Ayrapetyan et al., 2014). However, much more work is needed in order to understand the detailed mechanisms by which this gene and other genes induce the resuscitation of VBNC cells.

## Significances

### VBNC: changing the face of pathogen detection

More than 80 bacterial phyla have been identified to date; however, only about half of these have members that can be cultured in the laboratory (Pace, 2009; Stewart, 2012). Evidently, the history of microbiology has had a vested interest in microorganisms that have a direct influence on human health and food production, so most of the culturable species are either probiotics, pathogens, or those related to food spoilage. Until the discovery of the VBNC state (Xu et al., 1982), the presence and viability of these microorganisms were equated with their culturability on defined media. It is now increasingly clear that the potential threat presented by a given bacterial pathogen is not completely represented by its culturability on artificial media. Alternatives to culturing bacterial agents of disease use nucleic acid and antigen-based tests. While these are faster than culture methods, they are not without limitations making it worthwhile to invest energy into ameliorating current culture techniques (Cronquist et al., 2012; Jones and Gerner-Smidt, 2012). As discussed above, various VBNC-inducing conditions have been identified to date and these conditions are most likely just the tip of the iceberg. Research on the VBNC state is still in its infancy and in all likelihood, there are many more stresses that can induce a non-culturable state that are yet to be identified.

Entry into a VBNC state is considered as a bacterial response to exogenous stress. While these stresses may originate from the natural environment, what raises even more concern are the stresses induced by antimicrobial therapies and disinfectants used by humans to cure infections, obtain safe food and water sources, and sanitize environments. These may trigger bacteria to enter a VBNC state, especially when used inaccurately or in sub-lethal amounts. Noncompliance to drug regimens have been identified as a cause for the emergence of multidrug resistant strains of bacteria and this may well be a trigger for the VBNC state in some pathogens like *M. tuberculosis* (Kardas, 2002; Anderson et al., 2013).

More importantly, many documented VBNC inducing conditions are those in which humans encounter pathogens. For example, monochloramine treatment, which is used to sterilize drinking water, renders *L. pneumophila* VBNC, as does prolonged incubation in tap water (Steinert et al., 1997; Ducret et al., 2014). *M. tuberculosis* becomes VBNC under hypoxic conditions, theorized to be a stress encountered by the bacterium inside the tubercules of *M. tuberculosis* carriers (Fattorini et al., 2013; Manina and McKinney, 2013). *V. vulnificus*, a food-borne pathogen, enters the VBNC state in refrigerating temperatures (Nowakowska and Oliver, 2013).

Diagnosis of disease and identification of etiological agents have been and still are highly dependent upon culture techniques. The inability to culture microorganisms is then a major impasse for proper diagnosis of diseases and subsequent treatments, thus posing serious problems to pathogen detection, not only in the environment but also in food and water sources. As such, potentially dangerous contaminations can elude detection. In fact, VBNC bacteria have been found in human urine samples that were previously considered sterile (Anderson et al., 2001). Moreover, uncovering the secrets of the VBNC state may also provide clues to improve culture methods for the environmental bacteria that remain uncultivable as only 1% of the environmental bacterial population is thought to be cultured to date (Bloomfield et al., 1998). Giving the lack of novel antibiotics, enhanced culture techniques may lead to the discovery of new antimicrobial compounds.

### VBNC and biofilm

Biofilms are surface-attached, sessile bacterial communities enclosed in an extracellular matrix (ECM) (O'Toole et al., 2000). It has been estimated that 95% of bacteria present in water systems are found in biofilms (Flemming et al., 2002). Since water is a major source of contamination in food and an integral part of food production, and its contamination is the root of several nosocomial infections, focus on VBNC cells inside biofilms is of particular interest. While Kell and Young (2000) argue that the basis for the difference between dormancy and the VBNC state has been attributed to differences in metabolic activity, the literature suggests that both terms are describing the same physiological state of the cell. In the case of dormancy, the halt of cell division (leading to non-culturability) is viewed as a product of the dormant state while the VBNC state is defined by the inability of a cell to grow on laboratory media. Until our understanding of VBNC is deepened, this differentiation seems to be based on semantics and not necessarily on the actual presence of two different states.

Several reports show that the heterotrophic plate count of biofilms from water samples do not reflect the total cell counts and this difficulty has been attributed to a VBNC status achieved within the biofilm (Wingender and Flemming, 2004; Lee et al., 2007). To date, little focus has been placed on studying VBNC cells in biofilms, but the prevalence and the threats associated with biofilms in medical settings will likely result in more rigorous research into this area of biofilm study. For example, *C. jejuni*, a food-borne bacterium, forms VBNC cells in biofilms where biocide resistance is increased (Newell and Fearnley, 2003). *S. epidermidis* in biofilms was found to enter a VBNC state when grown in media with an excess of glucose (Cerca et al., 2011). Interestingly, these VBNC cells induced a lower production of the cytokines TNF-α and IL-6, and replicated in macrophages to a lesser extent than their culturable counterparts leading to lower macrophage death. While the authors conclude that VBNC cells lead to a lower activation of macrophages, the CFU counts were only taken up to 9 h post-infection, which may not have been sufficiently long enough for resuscitation inside the host cells. VBNC *Staphylococcus aureus* and *S. epidermidis* cells have also been isolated from biofilms inside catheters (Zandri et al., 2012). More specifically, Pasquaroli et al. (2013) found that *S. aureus* entered a VBNC state upon exposure to antibiotics. However, subsequent resuscitation in response to sodium pyruvate makes it unclear whether the VBNC cells reported were, in fact, injured cells. More recently, *L. monocytogenes*, another important food-borne pathogen, was also shown to produce VBNC cells in biofilms (Gião and Keevil, 2014).

The microenvironment inside the biofilm structure is subjected to oxygen, pH and nutritional stresses; there is a lower nutrient availability and higher waste concentration deeper inside the biofilm than in the periphery (Stewart and Franklin, 2008). It is well established that bacteria in biofilms are starved and this starvation response confers antibiotic resistance (Nguyen et al., 2011). Such bacteria are thought to be thousand times more resistant to antimicrobials than their planktonic counterparts (Costerton et al., 1999). In multispecies biofilms, the culturability of *L. pneumophila* is influenced by the presence of other species (Gião et al., 2011). It is clear that much more rigorous research needs to be conducted to study the formation and impact of VBNC cells in biofilms.

### *Mycobacterium tuberculosis*: an example of non-culturable cells *in vivo*

The entry into the VBNC state has been identified in response to simulated environmental stresses and there is evidence of resuscitation in response to co-culture with host cells or their supernatants (Senoh et al., 2012). *M. tuberculosis* is known to enter VBNC state and resuscitate during infection of the host (i.e., human lungs). It is also well established that once the selective pressures for latency is absent, mainly in the form of immunosuppression, these dormant bacteria can cause active tuberculosis (TB). The VBNC state of *M. tuberculosis* (Mtb) is also known as latency or dormancy, and contributes to over two million Mtb carriers around the world (Gengenbacher and Kaufmann, 2012).

Once it invades alveolar macrophages, *M. tuberculosis* can hijack host cell machinery to halt phagosome maturation (Crowle et al., 1991; Sturgill-Koszycki et al., 1994; Vergne et al., 2004), but *Mycobacterium* spp. have also been shown to endure the stresses of a mature phagolysosome (Via et al., 1998). Latent TB is the result of solid granulomas containing *M. tuberculosis*, which is considered as VBNC cells because of decreased culturability (Gengenbacher and Kaufmann, 2012; Reece and Kaufmann, 2012). When the bacterium is non-culturable inside the human lung and alveolar macrophages, it does not cause any diagnosable symptoms until reactivation. Both *in vitro* (Wayne model) and *in vivo* (Cornell model) models are used to generate VBNC cells of *M. tuberculosis* in order to study bacterial and host factors contributing to the VBNC status (Wayne and Hayes, 1996; Scanga et al., 1999).

In the past 20 years, a growing body of research has been investigating Rpfs in *M. tuberculosis* and related mycobacteria that are at the root of latent TB reactivation (see section Mechanisms of Resuscitation). Mtb-derived Rpfs have been shown to increase the culturability of VBNC bacteria in clinical sputum samples (Mukamolova et al., 2010). More recently, highly specific free fatty acids have been identified as playing a role in a regulatory cascade involving Rpf proteins and adenylate cyclase, leading to the resuscitation of *M. tuberculosis* cells (Shleeva et al., 2013). The use of resuscitation knowledge in treating latent TB infections may be the next step in eliminating or reducing the disease burden.

### Antibacterial resistance of VBNC cells

Because VBNC bacteria have a low metabolic rate, it is reasonable that antibacterials that target activities or components of active cells would be less effective against them. VBNC cells inside biofilms have an additional protection by the ECM. Moreover, the hypothesis that the VBNC state is an adaptation to stressful environments means that, by definition, these cells are likely to be less sensitive to exogenous stress. In *M. tuberculosis*, VBNC cells are relatively insensitive to isoniazid, which is an antibacterial drug targeting cell wall synthesis (involved in replication of active cell), but it is still used as a long-term therapy to treat the latent phase of disease (Manina and McKinney, 2013). The effectiveness of isoniazid in the long-term supports the idea that VBNC cells have in fact, adopted a slow metabolism. Therefore, the VBNC state may favor the development of drug resistance when strict drug regimens are not followed by allowing the bacterium to adapt before resuscitation. Oliver (2010) reviewed the antibiotic resistance in the VBNC state of some bacteria including *E. faecalis*, *E. coli*, *Haemophilus influenza*, *H. pylori* and *M. smegmatis*, all of which demonstrated an increased resistance to antibiotics. In addition, VBNC cells of *E. coli* are more resistant to sonication and that of *C. jejuni* are more heat resistant than non-VBNC cells (Klančnik et al., 2009; Zhao et al., 2013). Recently, an exponential phase cell culture and a VBNC cell culture of an environmental genotype strain of *V. vulnificus* were exposed to different challenges. It was found that the VBNC cells are more tolerant to a list of exogenous stresses, including high temperature (50°C for 1 h), ethanol (final concentration of 13% for 1 h), high salinity (*c*. 290 ppt for 2 h), oxidative stress (0.2 mM H_2_O_2_ for 1 h), acidity (pH 3 for 25 min), antibiotics (ampicillin or chloremphenical for 4 h) and zinc (3.4 mM ZnSO_4_7H_2_O for 1 h) (Nowakowska and Oliver, 2013). Interestingly, the same study demonstrated a lower resistance in VBNC cells of the clinical strain than those of the environmental strain, and proposed that these differences are based on the expression of certain genes (e.g., *relA* and *katG*).

### Virulence and VBNC in other pathogens

The recurrence of *M. tuberculosis* infections clearly proves that VBNC cells in the latent phase of the disease are able to retain or, in the least, regain their virulence potential upon resuscitation. In fact, since the VBNC state is a slow metabolic state, it would seem more likely that the observed virulence is a result of reactivation of the normal function of the cell (Oliver, 2010). In support of virulence maintenance in the VBNC state, VBNC *L. pneumophila* has been demonstrated to retain the capacity to infect the amoeba used for resuscitation, demonstrating virulence toward its natural host (Steinert et al., 1997; Al-Bana et al., 2013). Interestingly, another *L. pneumophila* strain showed continued production of virulence-related proteins in VBNC cells but could not be resuscitated in the same amoeba (Alleron et al., 2013). VBNC cells of *Vibrio* spp. have the ability to cause disease after resuscitation in their respective hosts (Baffone et al., 2003; Sun et al., 2008) and a microarray analysis of four *Vibrio* spp. revealed that a number of virulence and toxin genes were expressed in the VBNC state (Vora et al., 2005). VBNC *S. typhi* was also able to infect and cause disease in mouse models after resuscitation (Zeng et al., 2013). Much like the vibrios, *E. coli* O157:H7 was shown to produce Shiga-like toxins, but the toxin levels produced were dependent upon the age and the inducing condition of the VBNC cells (Liu et al., 2010).

These data on VBNC virulence suggests that, much like VBNC inducing stimuli and resuscitation factors, the expression and maintenance of virulence in different species of VBNC cells can vary greatly. *P. aeruginosa* is an important pathogen in terms of the disease burden and mortality associated with infection. It colonizes lung tissues of cystic fibrosis patients and is difficult to completely eradicate as it resides in biofilms in the lungs (Mulcahy et al., 2013). Given that VBNC cells can occur inside biofilms, relatively little progress has been made to study resuscitation factors of *P. aeruginosa* in this environment.

This capacity of resuscitation with no apparent loss of virulence potential evidently brings about concerns regarding the presence of VBNC bacteria not only in a medical context with respiratory pathogens like *M. tuberculosis*, *L. pneumophila*, and *P. aeruginosa* but also in relation to food safety (Dinu and Bach, 2011). In the case of *P. aeruginosa*, great strides toward reducing the bacterial load in CF patients could be made by inducing resuscitation of the VBNC bacteria inside the human host which would then be more susceptible to antibiotics. It is an avenue of research that is worth exploring.

## Conclusion

After decades of study, it is clear that the VBNC state is both an important tool for the survival of bacteria and a dangerous aspect of bacterial pathogens for the host. The knowledge about the VBNC state comes from research on a variety of bacteria and highlights the complexity of this mechanism of adaptation. What seems clear is that induction and resuscitation of the VBNC state are highly variable across bacterial species and in some cases, strains. However, the basic genetic mechanisms may share a common theme and further research into this field will help tie up the loose ends that exist in this area. The ability to avoid conditions that lead to resuscitation, or the development of drugs that induce resuscitation during antibiotherapy could have a major impact on the consequence of the VBNC state in chronic infectious diseases. Development of new, inexpensive methods to easily detect cells in the VBNC state is needed to increase food safety. In conclusion, the potential applications of VBNC research are significant to prevent food- and water-borne infections, and find new treatments to cure chronic bacterial infections.

### Conflict of interest statement

The authors declare that the research was conducted in the absence of any commercial or financial relationships that could be construed as a potential conflict of interest.
